# Changes in the vaginal microbiota associated with primary ovarian failure

**DOI:** 10.1186/s12866-020-01918-0

**Published:** 2020-07-29

**Authors:** Juan Wang, Jieying Xu, Qixin Han, Weiwei Chu, Gang Lu, Wai-Yee Chan, Yingying Qin, Yanzhi Du

**Affiliations:** 1 Center for Reproductive Medicine, Ren Ji Hospital, School of Medicine, Shanghai Jiao Tong University, 845 Lingshan Road, Shanghai, 200135 China; 2 Shanghai Key Laboratory for Assisted Reproduction and Reproductive Genetics, Shanghai, 200135 China; 3 The Chinese University of Hong Kong-Shandong University Joint Laboratory on Reproductive Genetics, School of Biomedical Sciences, The Chinese University of Hong Kong, Hong Kong, SAR China; 4 National Research Center for Assisted Reproductive Technology and Reproductive Genetics, The Key Laboratory for Reproductive Endocrinology of Ministry of Education, Shandong Provincial Key Laboratory of Reproductive Medicine, Center for Reproductive Medicine, Shandong Provincial Hospital, Shandong University, Jinan, 250021 China

**Keywords:** Vaginal microbiota, Primary ovarian failure, 16S rRNA, Pathogenesis, Female reproductive tract

## Abstract

**Background:**

Primary ovarian failure (POF) is defined as follicular failure in women of reproductive age. Although many factors are speculated to contribute to the occurrence of POF, the exact aetiology remains unclear. Moreover, alterations in the microbiome of patients with POF are poorly studied.

**Results:**

This study investigated the vaginal microbiota of 22 patients with POF and 29 healthy individuals. High-throughput Illumina MiSeq sequencing targeting the V3-V4 region of the 16S ribosomal RNA (rRNA) gene was used to evaluate the relationships between the vaginal flora and clinical characteristics of POF. Different from results of previous studies, we found that the diversity and richness of the vaginal flora of patients with POF was significantly different from those of healthy controls. Comparison of the vaginal flora of patients with POF with that of menopausal women revealed that the relative abundance of *Lactobacillus* was significantly reduced in the latter. A reduced abundance of *Lactobacillus* was furthermore associated with a lower pregnancy success rate. Of particular interest is that *L. gallinarum* especially appeared to be beneficially associated with reproductive-related indicators (FSH, E2, AMH, PRL) whilst *L. iners* appeared to have a detrimental effect. The result of the present study may enable the identification of microbiota associated with POF, however, further investigations of differences in the microbiota in the context of POF will enable a deeper understanding of the disease pathogenesis that involves modification of the vaginal microbiota.

**Conclusions:**

The present study identified the microbiota associated with POF. Further investigations on the differences in the microbiota in the context of POF will improve our understanding of the pathogenesis of the disease which involves modification of the vaginal microbiota.

## Background

Primary ovarian failure (POF) is defined as the failure of ovarian function in women aged < 40 years. Clinically, the condition is characterised by amenorrhea lasting 4 months or more, accompanied by oestrogen deficiency and elevated levels of gonadotropin [1, 2]. The prevalence of POF is 1% in women aged < 40 years [3, 4], and the incidence is increasing. Development of POF is associated with particular autosomal gene defects, autoimmune dysfunction, infection, and iatrogenic factors. Smoking, drinking, and nutritional factors may influence the age at which menopause occurs [2]. However, in nearly half the cases of POF, the causal factors remain unclear. Furthermore, the clinical manifestation differs between individuals, the most serious being gonadal dysplasia secondary to infertility—only 5% of patients with POF can conceive naturally. Patients with POF can also experience comorbidities including osteoporosis, dyslipidaemia, blood pressure fluctuations, and cardiovascular disease [5]. The clinical recommendations are to undergo in vitro fertilisation (IVF)-embryo transfer with egg donation based on hormone-replacement therapy (HRT) [6]. The complexity of POF and the decreasing age of patients experiencing POF indicates that research regarding the underlying mechanism of the disease is of high importance.

There is ample evidence indicating that the vaginal microbiota of women of childbearing age mainly comprises *Lactobacillus* species [7]. *Prevotella*, *Atopobium*, and *Gardnerella spp*. are associated with bacterial vaginosis (BV), and their presence in the vaginal microbiota may lead to preterm birth [8]. A number of studies have found a high incidence of BV in women undergoing IVF, and reported that this condition is associated with infertility [9, 10]. Women with a high relative abundance of *Gardnerella* and *Atopobium spp* in the vaginal microbiota have been reported to have poor IVF outcomes in terms of pregnancy [11]. When the vaginal microbiota is altered, the production of lactic acid will be changed, potentially leading to an increase in the secretion of inflammatory factors such as interleukin (IL)-6, IL-8, and tumour necrosis factor (TNF)-α, which activate the immune system and cause the body to be in a chronic inflammatory state. This affects the success rate of pregnancy [12, 13], highlighting the important roles of vaginal microbiota in the reproductive tract microbiome and maintenance of reproductive tract health in female [14].

Primary ovarian failure has been shown to have an autoimmune component. Proposed mechanisms of POF have suggested that viral, genetic, or other environmental stimuli may induce the expression of major histocompatibility complex (MHC) Class I and class II antigens in granulosa cells. These antigens are recognised by ovarian T cells, which respond by secreting lymphokines to stimulate macrophages to secrete more interferon (IFN)-γ that further increases the expression of MHC in ovarian granulosa cells, thus triggering humoral and cell-mediated autoimmune responses including secretion of IL-1 from macrophages and lymphocytes and acceleration of follicular atresia [15]. However, the vaginal epithelium has many innate immune protection mechanisms including the presence of tight junctions, antimicrobial peptides (AMPs) and mucus. In addition, immune cells such as γ- and δ-T cells, dendritic cells (DC) and macrophages are present below and between the epithelial cell layer of the vagina [16].

Class II antigen expression can be induced in patients with POF, and the in vitro expression of these antigens in granulosa cells is enhanced by the addition of IFN-γ to cell culture [17]. Vaginal microbiota has also been linked to female infertility via its effect on the concentration of various inflammatory factors in the plasma. Compared with women with normal fertility, the vaginal lavage fluid of infertile women has been found to increase the levels of inflammatory factors such as TNF-α and IFN-γ, and decreased the levels of IL-6 and IL-10 [18]. A number of studies have also shown that the proliferation of *Gardnerella* vaginalis associated with inflammatory response can be inhibited by *Lactobacillus*. By activating TLR-2 on the surface of monocytic THP-1 cells, *G. vaginalis* activates NF-κB to induce the secretion of large amounts of TNF-α [19, 20]. Similar increases in TNF-α level have also been reported in a study on a transgenic rat model of POF [21]. Therefore, it is important to study the vaginal microbiota of patients with POF during disease development.

This is the first study to use 16S rRNA gene sequencing to investigate the microbial communities of the vaginal microbiota of patients with POF compared with those of healthy women. Furthermore, we analysed the relationship between vaginal microbiota and clinical characteristics of POF.

## Results

### Demographic and clinical characteristics of the study population

We enrolled 22 women with POF and 29 healthy controls for analysis. The clinical characteristics of the two groups are shown in Table 1. Among patients with POF, the mean age was 30.50 ± 3.17 years, body mass index (BMI) was 22.34 ± 3.32 and waist-to-hip ratio was 0.83 ± 0.04. Among the healthy control group, the mean age was 29.79 ± 3.99 years, BMI was 23.47 ± 3.51 and waist-to-hip ratio was 0.84 ± 0.06. Age, BMI and waist-to-hip ratio were not significantly different between the two groups (*P* > 0.05), whereas AMH and E2 were significantly lower in the POF group (*P* < 0.001). Levels of FSH and LH were higher among patients with POF than among the control group (*P* < 0.001 and *P* < 0.01, respectively). Among menopausal women, the mean age was 57.96 ± 6.57 years, BMI was 23.57 ± 3.21, and menopause had been experienced for at least 1 year.

**Table 1:** Clinical information of patients

	Primary ovary failure	Control group	*P* value
Number of subjects	22	29	
Age(year)	30.50 ± 3.17	29.79 ± 3.99	> 0.05
Waist to hip ratio(cm)	0.83 ± 0.04	0.84 ± 0.06	> 0.05
BMI(kg/m^2^)	22.34 ± 3.32	23.47 ± 3.51	> 0.05
AMH(pmol/L)	0.06 ± 0.03	4.31 ± 2.25	< 0.001
FSH(IU/L)	75.28 ± 27.60	5.82 ± 1.22	< 0.001
LH(IU/L)	42.23 ± 16.00	4.68 ± 1.51	< 0.001
E2(pg/ml)	17.47 ± 19.75	35.56 ± 17.19	< 0.01

Data shown as mean ± SD. BMI, body mass index (kg/m2); Calculated using two independent samples T test

### Microbial profiling

**Fig. 1:**
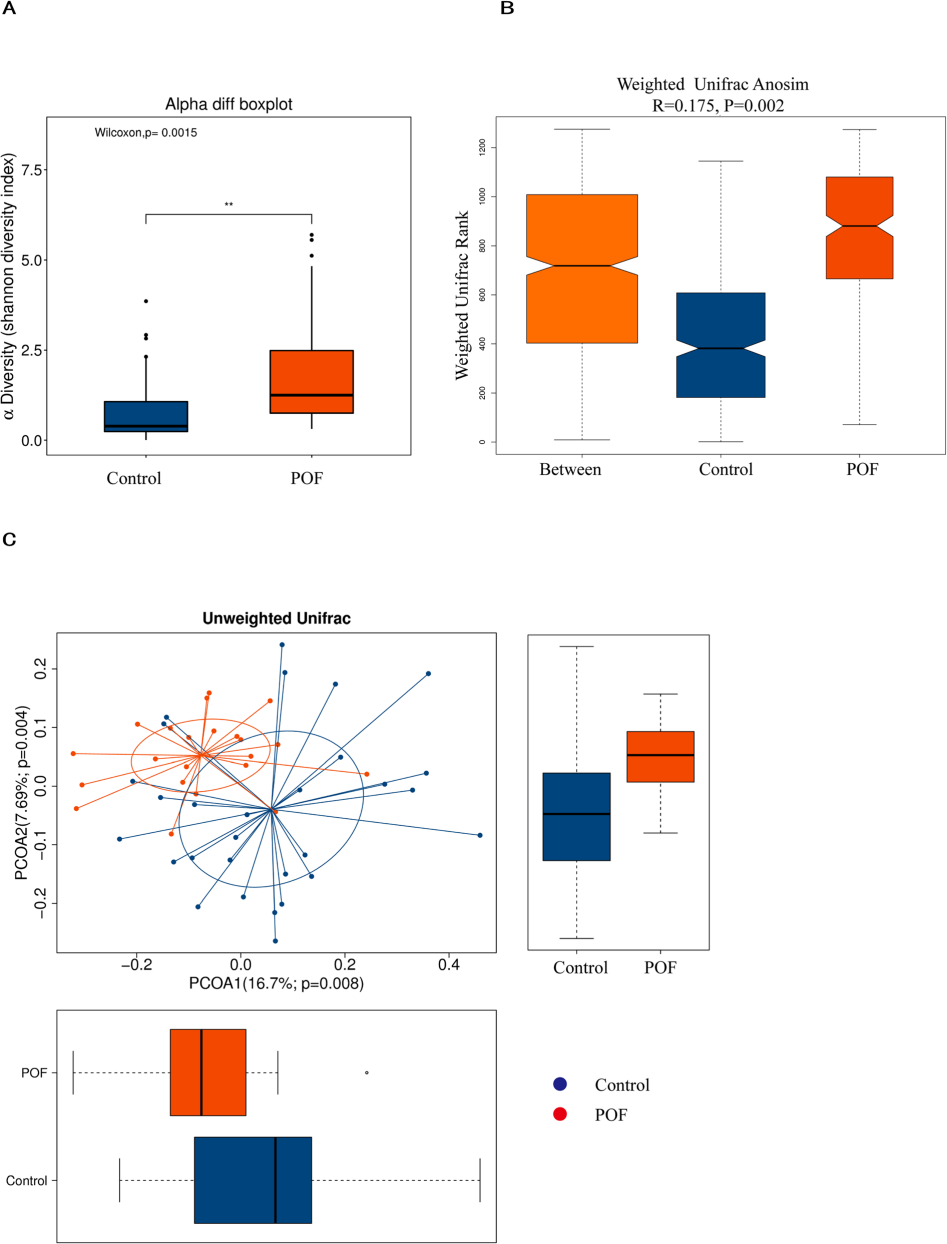
Comparison of diversity and shift of vaginal flora composition of females with POF and healthy controls. **a** The abscissa indicates the sample grouping, and the ordinate indicates the alpha diversity index value under different groupings. A greater Shannon value indicates higher diversity. **b** Beta diversity analysis is used to compare species diversity between each sample. The abscissa represents all samples (between) and each group, and the ordinate represents the rank of the Unifrac distance. R > 0 indicates that the between-group difference is greater than the within-group difference; R < 0 indicates that within-group difference is greater than the between- group difference. *P* < 0.05 was considered as statistically significant. **c** Horizontal and vertical coordinates represent the first and second main coordinates, respectively. Percentages indicate the contribution rate of the corresponding main coordinate to the sample difference, and the *P* value is the test *p* value of the corresponding main coordinate. The points represent the respective samples, different colours represent different groups. The horizontal box diagram illustrates the distribution of values of different groups on the first principal coordinate; the vertical box diagram illustrates the distribution of values of different groups on the second principal coordinate

The clean reads we obtained after quality control tend to be flat as the depth of sequencing increases (Supplementary Figure 1).The mean community diversity indexes (alpha diversity, including Chao1, observed species and Shannon and Simpson indices) were significantly higher in the POF group than in the control group (Fig. 1a, Supplementary Fig. 2, *P* < 0.01). Beta diversity was also significantly different between groups according to the weighted UniFrac phylogenetic distance matrices (analysis of similarities, *R* = 0.175, *P* = 0.002) and showed in PCoA plots (Fig. 1b and c). Thus, the vaginal microbiota of the POF group was significantly different to that of the control group. Detailed 16S rRNA raw sequence data are available in the NCBI Sequence Read Archive (SRA) under accession number PRJNA594533.

### Abundance of taxa in the two groups

**Fig. 2:**
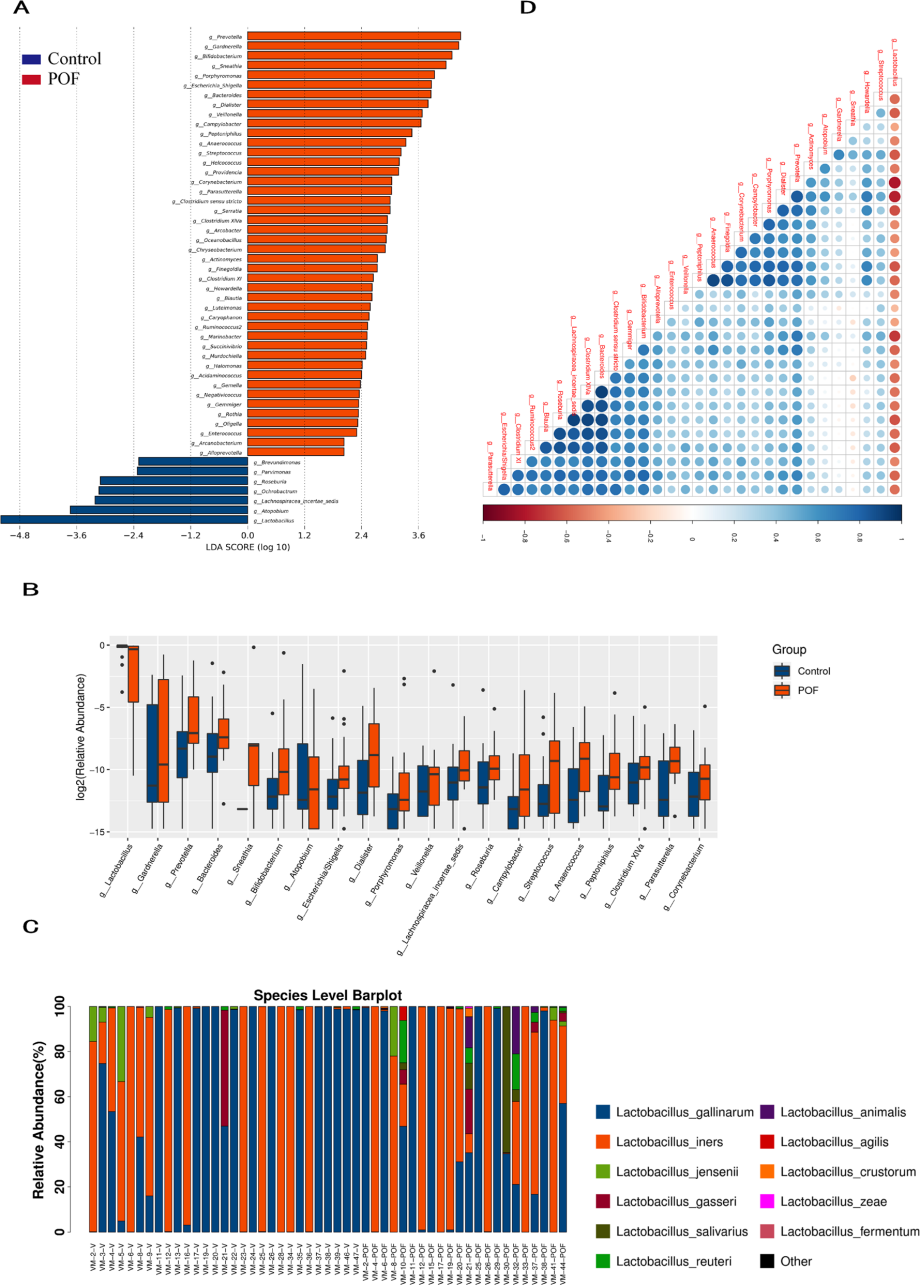
Comparison vaginal flora phylotype between groups. **a** Differential genera in vaginal microbiota between patients and control groups. **b** The abscissa is the name, the ordinate is the value of the log of relative abundance, and different colours represent different groups. Species that were abundant in at least one group are not displayed. **c** Species abundance map of the two groups. **d** The correlation coefficient between top 30 most abundant species at all levels of classification. Right blue indicates a positive correlation and red indicates a negative correlation. Darker colour indicates a stronger correlation between the species. The species prefixes “k__”, “p__”, “c__”, “o__”, and “f__” on the left indicate that the species are annotated to the boundaries, gates, classes, orders, and subjects

By LEfSe analysis, we identified 51 genera-discriminative features (Fig. 2a, LDA > 2, *P* < 0.05). Comparison of vaginal microbiota by Mann-Whitney U test revealed 51 taxa that were differentially abundant between the groups (*P* < 0.05); the species of the top 20 are shown in Fig. 2b. The agreement of results of the two analytical methods indicates the stability of the vaginal microbiological data.

The relative abundance of bacterial taxonomic groups at the genus level showed that 10 genera including *Gardnerella*, *Prevotella*, *Bacteroides*, *Sneathia*, *Dialister* and *Anaerococcus* were abundant in the POF group. Only *Lactobacillus* was found to be abundant in the control group (Supplementary Table 1). The *Lactobacillus* members are mainly grouped into the following two species: *L. gallinarum* (56.33%) was the most abundant, followed by *L. iners* (39.48%) and *L .jensenii* (2.13%) (Supplementary Table 2) in the control group. However, as per our observation in patients with POF, the relative abundance of *L. iners* (45.89%) increased whilst colonization with *L. gallinarum* (42.69%) and *L. jensenii* (1.37%) decreased (Fig. 2c).

A heatmap visualising the spearman rho correlation coefficients of the above-mentioned genera revealed that *Lactobacillus* was negatively correlated with all other genera (Fig. 2d). Various (non-vaginal) *Lactobacillus* species are used as probiotics in the gut as they appear to be beneficial in human health [22, 23]. but it can be imagined that additional research would lead to the use of specific vaginal *Lactobacillus* species being used as vaginal probiotics. In this study, *Lactobacillus* and *L. gallinarum* in particular appear to be protective against POF. Studying the interactions of this genus, or of specific *Lactobacillus* species, with other genera would be a first step in exploring this potential use.

### Analyses of correlations between reproduction-related clinical indicators and vaginal flora

**Fig. 3:**
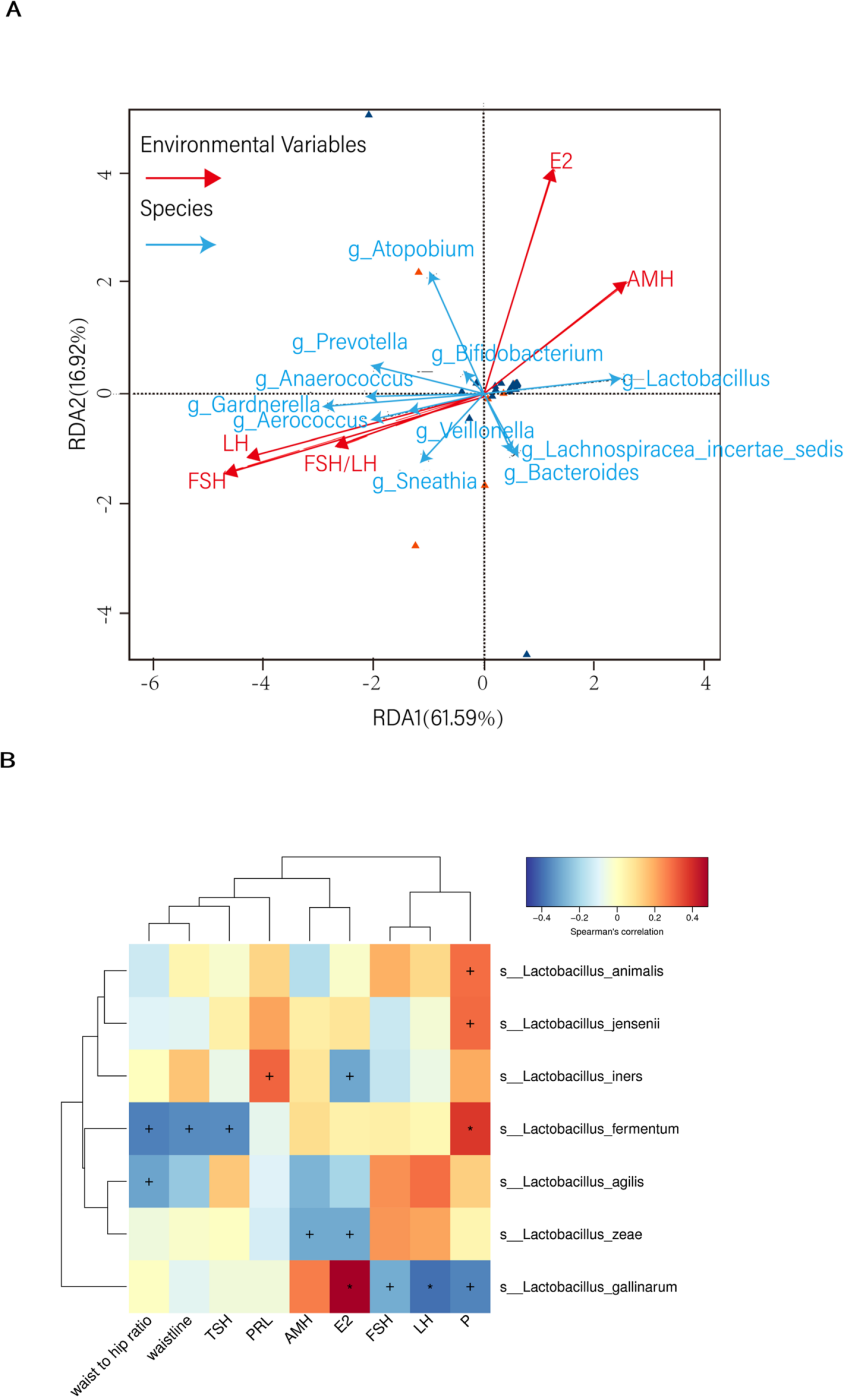
Coloured triangles represent sample groups in different environments or under different conditions. **a** red: POF group, blue: control group; arrows represent different reproductive-related indicators; an acute angle between arrows indicates a positive correlation, a negative correlation is indicated by an obtuse angle. The length of the solid line of the environmental factor indicates the impact of the factor. Dotted lines pointing to the type of bacteria indicate the corresponding genus level. **b** Hccc between reproductive-related indicators and *Lactobacillus* species. The abscissa indicates environmental factors, and the ordinate indicates species, the depth of the colour visually shows the correlation between the species and the environmental factors. When *P* < 0.05, “+” marks the significant. When *P* < 0.01, “*” marks significance

Redundancy analysis was used to produce a two-dimensional sorting map relating vaginal flora to reproduction-related clinical indicators. Serum FSH and LH levels showed the greatest association with female vaginal flora, and E2 had a significant effect. *Gardnerella* and *Prevotella* were positively correlated with serum FSH and LH levels, and negatively correlated with E2. *Lactobacillus* in the vagina was positively correlated with E2 and negatively correlated with serum FSH and LH levels. Similar to E2, AMH was positively correlated with *Lactobacillus* and negatively correlated with *Gardnerella* and *Prevotella* (Fig. 3a). Subsequently, we analysed the correlation between the *Lactobacillus* members and clinical indicators. The relative abundance of *L. gallinarum* was negatively corrected with FSH, LH and P levels. Meanwhile, the abundance of *L. gallinarum* was positively related to E2 level and there was a significant difference (*P* < 0.05). In addition, the relative abundance of *L. iners* was negatively related to E2 level indicating that *L. gallinarum* and *L. iners* play a weighted role in POF patients (Fig. 3b).

Next, the random forests model was analysed based on the vaginal flora profile including the taxon that exhibited significantly different abundances at the genus level. We identified 34 genera that could be used to predict occurrence of POF with the random forests model (Supplemental Fig. 3A). A mean classification error of 0.382 was achieved, and the AUC was 0.841 (95% confidence interval [CI]: 0.618–1, sensitivity: 71.4%, specificity: 100%, cut-off rate: 43.2%; Supplemental Fig. 3B).

### Functional alterations in the vaginal flora

Thereafter, we analysed the metabolic pathways of the two groups of subjects. The predicted genome database has been greatly expanded such that related enzymes, genes and other information can be obtained in addition to metabolic pathways. LEfSe analysis identified 17 KOs identifiers enriched in patients with POF. Including K02014 (iron complex outermembrane recepter protein), K07497 (putative transposase), K00123 (formate dehydrogenase major subunit), K00799(glutathione S-transferase) and K01223 (6-phospho-beta-glucosidase) (Supplemental Fig. 4A). KOs represent functional orthologs in the context of KEGG pathway maps and are defined by extending experimental knowledge in specific organisms to other organisms [24].

Supplemental Fig. 4B shows the results of Metacyc, the predictive functions performed using PICRUSt2.0. The pathways that were significantly enriched in POF were gondoate biosynthesis, fatty acid elongation – saturated, palmitoleate biosynthesis I (from (5Z)-dodec-5-enoate), superpathway of glycolysis, pyruvate dehydrogenase, TCA, and glyoxylate bypass, superpathway of tetrahydrofolate biosynthesis and salvage, pyridoxal 5′-phosphate biosynthesis I, superpathway of pyrimidine deoxyribonucleotides de novo biosynthesis, D-galactarate degradation I and so on. Whereas the microbial functions related to aerobic respiration I (cytochrome c), myo-, chiro- and scillo-inositol degradation, adenosine nucleotides degradation II, superpathway of L-serine and glycine biosynthesis I were higher in the vaginal microbiota of the POF group (Supplemental Table 3).

### Comparison of vaginal flora in the case of premature ovarian failure or menopause

**Fig. 4:**
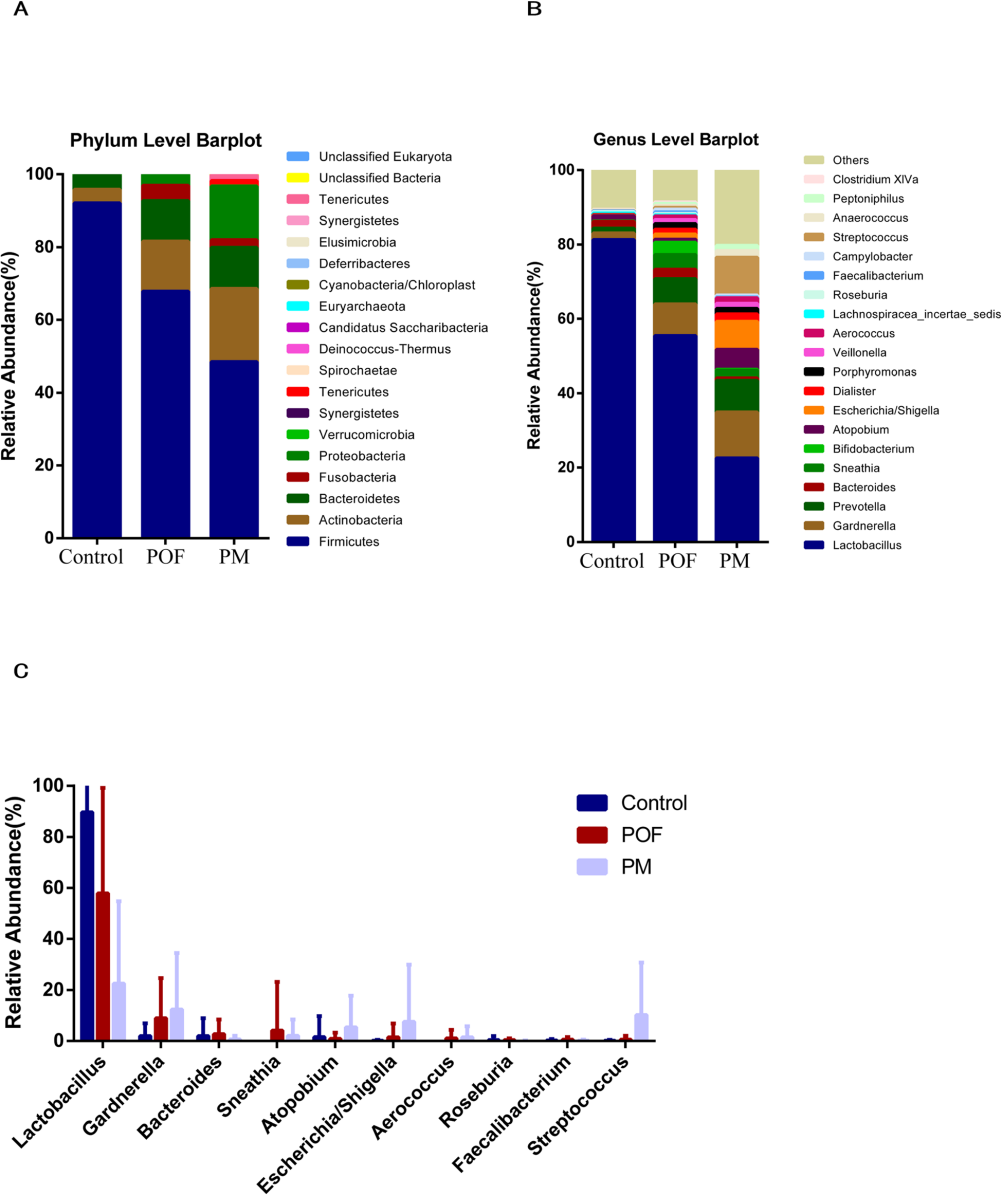
Species abundance map between POF with menopause. **a** Phylum level barplot; **b** Genus level barplot; **c** Species of significant differences between Control group, POF and menopause

Finally, we compared the vaginal microbial composition of patients with POF and menopausal women. The high abundance of *Lactobacillus*, *Gardnerella* and *Prevotella* was confirmed on the basis of a comparison of vaginal microbiota of patients with POF and menopausal individuals. However, the vaginal flora of menopausal women exhibited increased diversity (Fig. 4a and b). Differential species analysis showed *Lactobacillus* to be less abundant among menopausal women than among patients with POF (Fig. 4c).

## Discussion

In recent years, a wealth of evidence has been published supporting the significant contribution of cervicovaginal microbiota to genitourinary and reproductive health outcomes [25]. It was first found that the microbial taxonomic composition differs between patients with POF and healthy individuals. We found the vaginal microbiota to be increasingly diverse with increased species richness in case of patients with POF, and a significant shift in overall microbial diversity was observed. However, a previous cross-sectional study of microbiota failed to identify obvious differences between individuals in terms of vaginal microbiota diversity [26, 27]. The strength of our study lies in the comprehensive description of microbial communities associated with POF achieved through the use of 16S rRNA sequencing; particularly, the association with clinical characteristics of POF; and the utilisation of predictive models to identify bacterial taxa that are differentially expressed in POF.

Previous studies on the vaginal microbiota in patients with POF have mainly involved the amine test (or the Whiff test) [26], whereas our study focused on differences at the genus level. One of the most attractive features of 16S rRNA gene sequence informatics is the potential for genus and sometimes even species identification [28]. Dysbiosis of the vaginal microbiota was characterised by the altered abundance of 34 genera in POF. The combination of these 34 associated taxa enabled the differentiation of patients with POF and the control group with high accuracy. We noted that vaginal-microbiota-based analysis displayed a similar predictive ability for the disease as the classifier based on POF-associated genetic variants (with an AUC of 0.841, sensitivity of 71.4%, specificity of 100% and cut-off rate of 43.2%), implying that the microbial signature that we identified could represent a powerful tool for the prediction of POF. Our results of the changes in the relative abundance of a particular genus in terms of POF confirm that *Lactobacillus* is the dominant vaginal genus. In most women in China *L. iners* and *L. gallinarum* are the dominant facultative anaerobes of the genital tract [29, 30].

The abundance of *Lactobacillus* was lower in the vaginal microbiota of women with higher levels of basal FSH and/or lower levels of basal E2. CR et al. evaluated the presence of *Lactobacillus* spp. in confluent monolayers of endocervical, ectocervical, and vaginal epithelial cells, they found that the *Lactobacillus spp* can stimulate significant cytokine and AMPs induction [31]. The expression of AMPs, which include cathelicidins and defensins, can also promote IL-22 secretion and thus prevent autoimmune diseases [32]. A previous study suggested that *Lactobacillus* can reinforce the mononuclear phagocytic response by inducing production of the autophagy-promoting factors [33]. Studies have also shown that inflammatory ageing and autoimmune responses are closely related to POF [15, 34]. Our results suggest that reduced colonization of *Lactobacillus* may accelerate the development of POF through the induction of immune responses by some inflammatory factors.

Moreover, our study found for the first time that the relative abundance of *L. gallinarum* in the vagina was correlated with the FSH, E2 and AMH levels. FSH and AMH levels were previously thought to be exclusively regulated by the ovaries [35, 36]. Moreover, we showed that *L. gallinarum* was positively correlated with E2 level, *L. iners* was negatively correlated with E2 level, *L. iners* is furthermore positively associated with PRL, which was consistent with our clinical changes. PRL is a hormone that plays a role in fertility by inhibiting follicle stimulating hormone (FSH) and gonadotropin-releasing hormone (GnRH) [37], the hormones that trigger ovulation and allow follicles to develop and mature. Hence, we’d think that more PRL may lead to hypogonadism and infertility in females. That means the decrease of the relative abundance level of *L. gallinarum* and the increase of the relative abundance level of *L. iners* might be associated with the decline of ovarian function. However, According to previous studies, the role of *L. iners* is contextual in different populations and the clinical conditions of HPV infection [38, 39]. Therefore, its effect on the host warrants further study under a variety of health conditions.

As previously studied, hormonal changes cause menstruation and menopause, resulting in a considerable reduction in the amount of *Lactobacillus* in the vaginal microbiota. In this case, infections caused by *Gardnerella vaginalis* are increased. *Gardnerella vaginalis* plays a significant role in vaginal immunity. In fact, the overgrowth of anaerobic species during menopause can increase the release of immune molecules such as NF-κB, TNFα, COX-2 and iNOS [40]. In addition, we observed higher levels of the genera *Prevotella* and *Gardnerella.* Abnormal vaginal microbiota may adversely affect the health of a pregnant woman. We observed the negative correlation between anaerobic species and *Lactobacillus*. These bacteria exploit the same class of environmental resources in a similar manner and are defined as an ecological “guild” [41]. Guild members do not necessarily share taxonomic similarities, but they adapt to the changing environment to co-exist and thus affect female reproductive function by altering the concentration of inflammatory factors.

Our functional analysis showed that the pathways involved in glycolipid metabolism and energy synthesis were related to POF [42]. However, limited information is available about the relationship between patients with POF and metabolic pathways based on the recent research on POF. According to the numerous results of KOs, we proposed a hypothesis of the underlying mechanisms on how imbalanced vaginal microbiota contributed to the pathological progress of POF (Supplemental Fig. 5). According to the hypothesis, enriched KOs indicate that POF patients harboured an impaired inflammation condition. Phosphatidylglycerol, phosphate, L-methionine and pyruvate, whose metabolic enzymes were enriched in the POF group, are known to be related to inflammation and Class II antigen [43, 44]. In turn, inflammation promotes the production of acetyl-CoA. Moreover, the excessive production of pyruvate may mediate mitochondrial dysfunction due to higher glucose environment and lead to cell apoptosis [45]. DNA damage activates the mitochondrial apoptosis pathway through oxidative stress, resulting in reproductive dysfunction [46]. In our experiment, glycolipid metabolism and some metabolic enzymes were enriched in the POF group. Moreover, the mTOR signalling pathway, whose definition is serine/threonine-protein kinase mTOR,[EC:2.7.11.1] was enriched in the control group, that was associated with autophagy (Supplementary Table 4).

When we compared the vaginal microbiota of menopausal women with that of women with POF, we found that although the three most abundant genera were the same, the relative abundance of *Lactobacillus* was reduced in menopausal women, which supports the findings of previous research [47]. It is well known that menopause and POF have similar clinical manifestations. Menopause is a natural physiological phenomenon caused by age, but POF is mostly related to genetics and immunity. Our results further validate the important role of the relative abundance of *Lactobacillus* during the development of ovarian insufficiency.

In conclusion, our study provides a clear link between POF, vaginal *Lactobacilli* and what is in literature known as community type IV (REFS). Of the *Lactobacilli, L. iners* is commonly also associated with various of the community type IV members (such as *Gardnerella, Prevotella*, etc. (REF)) and was strongly positively associated with PRL levels. On the other hand, *L. gallinarum* appears to be an especially promising candidate for further studies as it was for example strongly positively correlated with E2 but was also associated in various way with other hormonal levels. Future treatments of POF could perhaps aim to alter the microbiota of the reproductive tract using specific Probiotics (“vagina-appropriate” Lactobacillus species) in an effort to slow down follicular atresia, hence potentially improving the success rate of IVF. However, the present study had several limitations which should be addressed in future studies. First, the sample size was small and we could not trace the date of the POF diagnosed. Second, we could not clarify detailed roles of specific constituents of the vaginal microbiota in the pathogenesis of POF. Third, the inclusion of a disease verification model could reveal more accurate information related to the composition of the microbiome and its functions. Therefore, future studies involving larger study populations and animal models are needed to explore potential mechanisms underlying the association of the vaginal microbiota and POF. Genomics represents a potential approach to elucidate associations between the vaginal microbiota and disease, and analysis of the gut microbiota may help to explain other pathologies and improve many aspects of prevention and treatment.

## Conclusions

The result reveals for the first time that there are differences in the reproductive tract flora of women with premature ovarian failure, confirming that *Lactobacillus* plays a vital role in female reproductive health. We suggest that *Lactobacillus* may affect women’s ovarian function via inflammation and mitochondrial dysfunction. Future research in the future will provide new possibilities for the treatment of POF.

## Methods

### Study cohort and sample collection

We recruited 22 patients aged 20–40 years who visited the Reproductive Hospital affiliated to Shandong University for POF women from June to August 2018. According to the diagnostic criteria for POF [48], patients reported a previously regular menstrual cycle with the cessation of menstruation for at least four menstrual cycles and a serum follicle-stimulating hormone (FSH) level of 40 IU/L for more than 1 month. All patients with POF received hormone replacement therapy for more than 3 consecutive months, but serum hormone levels still met the diagnostic criteria for POF. We also recruited 29 healthy volunteers in the control group. These participants were selected according to normal menstrual cycles and regulatory factors (FSH of ≤10 IU/L, anti-Mullerian hormone (AMH) of ≥2 IU/L). Exclusion criteria for both groups were antibiotic treatment within 3 months prior to enrolment, liver or kidney dysfunction, surgical resection of one side of the ovary, previous smoking history, and vaginal medication in the past 3 days. In addition, this study included 50 women with standard post-menopausal data for at least 1 year and excluded those who had other organic lesions. This study and all its protocol were approved by the Reproductive Ethics Committee of Ren Ji Hospital affiliated to Shanghai Jiao Tong University School of Medicine (approval number: 2018072610).

The outpatient doctor at the department of gynaecology collected vaginal secretions from the vaginal posterior fornix using a sterile cotton swab according to standard clinical practice. Samples were treated by adding 750 μl of PowerSoil®-htp Bead Solution (MO BIO Laboratories, Inc. Carlsbad, CA, USA; catalogue number 12955–12-BS) and then stored at − 80 °C until analysis. Samples were collected from the posterior vaginal fornix and were stored in duplicate.

### Laboratory measurements

Baseline blood samples were collected and stored at − 80 °C until measurement. Serum AMH, FSH, luteinising hormone (LH), estradiol (E2) and thyroid stimulating hormone (TSH) were tested used enzyme-linked immunosorbent assay (ELISA) in the laboratory.

### Extraction of DNA and 16S rRNA amplicon sequencing

We isolated DNA from vaginal samples and assessed DNA quality using a Thermo NanoDrop 2000 UV spectrophotometer and electrophoresis on 1% agarose gel to assess DNA integrity and size. The 16S rRNA gene was amplified using the universal primers U341F 5′- CCTACGGGRSGCAGCAG − 3′ and U806R 5′-GGACTACVVGGGTATCTAATC − 3′ targeting the V3-V4 hypervariable regions. All quantified amplicons were pooled to equalise concentrations prior to sequencing using the Illumina MiSeq system (Illumina Inc., CA, USA). Library construction and sequencing were conducted at the Realbio Genomics Institute (Shanghai, China). Reads obtained by paired-end sequencing were merged into a sequence by the Pandaseq 2.9 [49] to obtain long reads of the hypervariable region. Thereafter, we performed the following processing on the stitched Reads to obtain Clean Reads: remove the Reads with an average quality value of less than 20, remove the Reads with more than 3 N-based bases and Reads range is 220 ~ 500 nt. To facilitate downstream microbial diversity analysis, long reads were clustered into operational taxonomic units (OTUs). Usearch V9 was used to cluster reads at a similarity of 0.97, then Chimeric sequences were removed to obtain OTUs [50], each of which was considered to represent a single taxon. We rarified our OTU table to 27,420 per sample, to minimise spurious effects of efforts on downstream diversity analyses. The most abundant sequence was selected from each OTU as a representative sequence for that OTU and was submitted to the Ribosomal Database Project (RDP) Classifier [51] to obtain the annotation. The annotated sequences were classified for each OTU and the composition of flora analysed at the level of the phylum, class, order, family, and genus. Based on the V3-V4 region (the length of the amplified high-variation region is limited), species-level annotation is usually not allowed. The reason why *Lactobacillus* can be analysed at the species level is because of the additional construction of the hidden Markov models (HMM). Using the software HMMER version 1.8.5, the models were built for each of the 42 known species of the genus *Lactobacillus*. Each V2 region of 16S rRNA gene sequences that assigned to the genus *Lactobacillus* was aligned to all species-level HMM models. If the highest HMM alignment score came from the i-th HMM model and that score was as high as the lowest score of the sequences used to build the i-th model, a read would assigned to the i-th HMM model. Using the DBSCAN clustering algorithm classified the sequence reads not assigned to any HMM model as OTUs within the genus *Lactobacillus* and align the HMM models of the five most abundant species in the dataset [52].

The bioinformatics pipeline QIIME1.9.1 [53] was used to calculate alpha diversity, perform beta diversity analysis and create the corresponding dilution curves. Alpha diversity represents the richness and evenness of microbial communities, including the observed species, Chao1, Shannon and Simpson indices and the phylogenetic diversity whole tree index. Beta diversity analysis is used to identify differences between different samples. To further investigate differences in taxon diversity between samples, we calculated the unweighted and weighted UniFrac distances for beta diversity analysis using the OTU table and phylogenetic tree. We generated ordination plots using principal coordinate analysis as implemented in R 3.5.1. Principal coordinate analysis (PCoA) was then performed, and linear discriminant analysis (LDA) effect size (LEfSe1.0) analysis [54] which is often used to identify the presence and effect size of region-specific OTUs among different groups was used to determine the microbiota associated with POF by comparing the flora of the POF and control groups [55]. The organisms that most comprehensively demonstrated the differences between groups were identified in different organisms using an LDA score cut-off of 2.0.

### Redundancy analysis

Redundancy analysis (RDA) is a multivariate direct gradient analysis method based on the development of corresponding analysis. Corresponding analysis is combined with multiple regression analysis and each step is calculated considering environmental factors. This analysis is based on a linear model and is mainly used to investigate the relationship between microflora and environmental factors. Network diagrams are drawn to determine the important relationship between species and environmental factors using Cytoscape [56].

### Random forest classification

Random forest classification is a tree-based algorithm that requires simulation and iteration and is utilised in machine learning. In general, random forests randomly generate hundreds to thousands of classification trees and select the tree with the highest degree of repetition as the final result. Based on the real category and prediction probability of the sample, receiver operating characteristic (ROC) curves can be generated, and the area under the curve (AUC) can be calculated to evaluate the model.

### Functional inference of 16S data

Functional annotation analysis was performed with PICRUSt2.0 (Phylogenetic Investigation of Communities by Reconstruction of Unobserved States) [57]. This software predicts functional abundance based on 16S rRNA genes sequence data and other marker gene reference sequence databases (covering KOs, EC, COG, MetaCyc, PFAM, TIGRFAM database, etc) to predict macro genomic functional composition. For accuracy, it is first necessary to standardise the number of genus of the original 16S sequencing data because the number of 16S copies present in different genus bacteria is different. The 16S genus composition information is then obtained by mapping the genogenic functional gene composition of the constructed sequenced genome to obtain the predictive functional results.

### Statistical analyses

Data analysis was performed using SPSS 23.0 statistical software. Normally distributed data sets were compared using independent-samples t test. Non-normally distributed data were compared using Wilcoxon signed-rank test function of the R language stats package. Continuous data are presented as mean ± standard deviation. *P* < 0.05 was considered statistically significant. Correlation analysis was performed using Spearman’s rank correlation coefficient.

## Supplementary information

**Additional file 1: Table S1.** Genera abundance.

**Additional file 2: Table S2.** Per-sample Lactobacilli species abundance.

**Additional file 3: Table S3.** MetaCyc annotation.

**Additional file 4: Table S4.** EC annotation.

**Additional file 5: Figure S1.** Diagram of the number of clean reads randomly selected from a sample, showing species diversity within each single sample. Sufficient sequencing depth of each sample is visualized by each of the curves becoming flat. Red indicates POF Groups; Blue indicates Control Group.

**Additional file 6: Figure S2.** The abscissa indicates sample grouping, and the ordinate indicates the alpha diversity index value under different groupings. The Chao1 index was used to estimate the total number of OTUs contained within a sample. Observed_ indicates the actual number of OUT observed. Greater Simpson value, higher diversity. Key: *0.01 < *p* < 0.05, **p < 0.01, “NS” indicates no significant difference.

**Additional file 7: Figure S3.** The predictive model based on genus-level abundance taxa using a random forests model. A: The difference in contributions of different species enabled groups A and B to be distinguished; B: The ROC curve of a random forest model was constructed based on the sorted different species, where the abscissa is 1-specificity and the ordinate is sensitivity. When the area under the curve (AUC is 0.5–0.7, the accuracy is low; when AUC is 0.7–0.9, there is certain accuracy; when AUC is above 0.9, the accuracy is high). Larger AUC indicates better model prediction effect.

**Additional file 8: Figure S4.** Functional predictions of vaginal flora of the POF and control groups. A: The abscissa is the log value obtained after KO has a significant effect in different groupings through LDA, the threshold for LDA was 2. Different colours represent that the EC is enriched in different groups of samples. B: The abscissa is the log value obtained after MetaCyc_pathway has a significant effect in different groupings through LDA, the threshold for LDA was 2. Different colours represent that the MetaCyc_pathway is enriched in different groups of samples.

**Additional file 9: Figure S5.** This diagram stands for a hypothesis regarding the possible mechanisms underlying relationship between vaginal microbiota abundance and pathological changes of POF. Gray text boxes denote enriched microbes, product, or pathway in POF patients. Red text boxes denote the pathological changes and complications in POF patients.

## Data Availability

The detailed 16S rRNA raw sequence data were available in the NCBI Sequence Read Archive (SRA) under accession number PRJNA594533.

## Abbreviations

P0F: Primary ovarian failure
AMH: anti-Müllerian hormone
FSH: follicle-stimulating hormone
LH: luteinizing hormone
E_2_: estrogen
PRL: prolactin
IVF: in vitro fertilisation
HRT: hormone-replacement therapy
KEGG: Kyoto Encyclopedia of Genes and Genomes
KO: KEGG orthologue
RDP: Ribosomal Database Project
HMM: hidden Markov models

## References

[1] Welt CK (2008). Primary ovarian insufficiency: a more accurate term for premature ovarian failure. Clin Endocrinol.

[2] De Vos M, Devroey P, Fauser BC (2010). Primary ovarian insufficiency. Lancet.

[3] Skillern A, Rajkovic A (2008). Recent developments in identifying genetic determinants of premature ovarian failure. Sex Dev.

[4] Altuntas CZ, Johnson JM, Tuohy VK (2006). Autoimmune targeted disruption of the pituitary-ovarian axis causes premature ovarian failure. J Immunol.

[5] Podfigurna-Stopa A (2016). Premature ovarian insufficiency: the context of long-term effects. J Endocrinol Investig.

[6] Sadeghi MR (2013). New hopes for the treatment of primary ovarian insufficiency/premature ovarian failure. J Reprod Infertil.

[7] Jang SJ (2019). Vaginal lactobacilli inhibit growth and hyphae formation of Candida albicans. Sci Rep.

[8] Li J (2012). Importance of vaginal microbes in reproductive health. Reprod Sci.

[9] Wilson JD, Ralph SG, Rutherford AJ (2002). Rates of bacterial vaginosis in women undergoing in vitro fertilisation for different types of infertility. Bjog.

[10] Eckert LO (2003). Relationship of vaginal bacteria and inflammation with conception and early pregnancy loss following in-vitro fertilization. Infect Dis Obstet Gynecol.

[11] Haahr T (2016). Abnormal vaginal microbiota may be associated with poor reproductive outcomes: a prospective study in IVF patients. Hum Reprod.

[12] Joo HM (2011). Lactobacillus johnsonii HY7042 ameliorates Gardnerella vaginalis-induced vaginosis by killing Gardnerella vaginalis and inhibiting NF-κB activation. Int Immunopharmacol.

[13] Rizzo A (2015). Lactobacillus crispatus mediates anti-inflammatory cytokine interleukin-10 induction in response to chlamydia trachomatis infection in vitro. Int J Med Microbiol.

[14] Koedooder R (2019). Identification and evaluation of the microbiome in the female and male reproductive tracts. Hum Reprod Update.

[15] Hill JA (1990). Induction of class II major histocompatibility complex antigen expression in human granulosa cells by interferon gamma: a potential mechanism contributing to autoimmune ovarian failure. Am J Obstet Gynecol.

[16] Nguyen PV (2014). Innate and adaptive immune responses in male and female reproductive tracts in homeostasis and following HIV infection. Cell Mol Immunol.

[17] Weiss G (2009). Inflammation in reproductive disorders. Reprod Sci.

[18] Ozkan ZS (2014). What is the impact of Th1/Th2 ratio, SOCS3, IL17, and IL35 levels in unexplained infertility?. J Reprod Immunol.

[19] Vick EJ (2014). Gardnerella vaginalis triggers NLRP3 inflammasome recruitment in THP-1 monocytes. J Reprod Immunol.

[20] Zariffard MR (2005). Induction of tumor necrosis factor- alpha secretion and toll-like receptor 2 and 4 mRNA expression by genital mucosal fluids from women with bacterial vaginosis. J Infect Dis.

[21] Fu Y (2012). Therapeutic mechanisms of Tongmai Dasheng tablet on tripterygium glycosides induced rat model for premature ovarian failure. J Ethnopharmacol.

[22] Nilsson AG (2018). Lactobacillus reuteri reduces bone loss in older women with low bone mineral density: a randomized, placebo-controlled, double-blind, clinical trial. J Intern Med.

[23] Malik M (2018). Lactobacillus plantarum 299v supplementation improves vascular endothelial function and reduces inflammatory biomarkers in men with stable coronary artery disease. Circ Res.

[24] Kanehisa M (2016). KEGG as a reference resource for gene and protein annotation. Nucleic Acids Res.

[25] Anahtar MN (2018). Cervicovaginal microbiota and reproductive health: the virtue of simplicity. Cell Host Microbe.

[26] Benetti-Pinto CL (2015). Vaginal epithelium and microflora characteristics in women with premature ovarian failure under hormone therapy compared to healthy women. Arch Gynecol Obstet.

[27] Pacello PC (2014). Dyspareunia and lubrication in premature ovarian failure using hormonal therapy and vaginal health. Climacteric.

[28] Janda JM, Abbott SL (2007). 16S rRNA gene sequencing for bacterial identification in the diagnostic laboratory: pluses, perils, and pitfalls. J Clin Microbiol.

[29] Younes JA (2018). Women and their microbes: the unexpected friendship. Trends Microbiol.

[30] Kroon SJ, Ravel J, Huston WM (2018). Cervicovaginal microbiota, women's health, and reproductive outcomes. Fertil Steril.

[31] Eade CR (2012). Identification and characterization of bacterial vaginosis-associated pathogens using a comprehensive cervical-vaginal epithelial coculture assay. PLoS One.

[32] Miani M (2018). Gut Microbiota-Stimulated Innate Lymphoid Cells Support β-Defensin 14 Expression in Pancreatic Endocrine Cells, Preventing Autoimmune Diabetes. Cell Metab.

[33] Ghadimi D (2010). Lactic acid bacteria enhance autophagic ability of mononuclear phagocytes by increasing Th1 autophagy-promoting cytokine (IFN-gamma) and nitric oxide (NO) levels and reducing Th2 autophagy-restraining cytokines (IL-4 and IL-13) in response to mycobacterium tuberculosis antigen. Int Immunopharmacol.

[34] Huang Y (2019). Inflamm-aging: a new mechanism affecting premature ovarian insufficiency. J Immunol Res.

[35] Sowers MR (2008). Follicle stimulating hormone and its rate of change in defining menopause transition stages. J Clin Endocrinol Metab.

[36] Freeman EW (2012). Anti-mullerian hormone as a predictor of time to menopause in late reproductive age women. J Clin Endocrinol Metab.

[37] Donato J, Frazão R (2016). Interactions between prolactin and kisspeptin to control reproduction. Arch Endocrinol Metab.

[38] Usyk M (2020). Cervicovaginal microbiome and natural history of HPV in a longitudinal study. PLoS Pathog.

[39] Lee JE (2013). Association of the vaginal microbiota with human papillomavirus infection in a Korean twin cohort. PLoS One.

[40] Quaranta G, Sanguinetti M, Masucci L (2019). Fecal microbiota transplantation: a potential tool for treatment of human female reproductive tract diseases. Front Immunol.

[41] D. Simberloff, T.D., The guild concept and the structure of ecological communities*.* Annu. Rev. Ecol. Syst., 1991. 22: 115–143.

[42] Chen W (2015). Low expression of Mfn2 is associated with mitochondrial damage and apoptosis of ovarian tissues in the premature ovarian failure model. PLoS One.

[43] Hough KP (2018). Unique lipid signatures of extracellular vesicles from the Airways of Asthmatics. Sci Rep.

[44] Abram QH (2018). Characterization of cDNA clones encoding major histocompatibility class II receptors from walleye (Sander vitreus). Mol Immunol.

[45] Feng J (2019). Mitochondrial pyruvate carrier 2 mediates mitochondrial dysfunction and apoptosis in high glucose-treated podocytes. Life Sci.

[46] Liu J (2018). Silica nanoparticle exposure inducing granulosa cell apoptosis and follicular atresia in female Balb/c mice. Environ Sci Pollut Res Int.

[47] Muhleisen AL, Herbst-Kralovetz MM (2016). Menopause and the vaginal microbiome. Maturitas.

[48] Shelling AN (2010). Premature ovarian failure. Reproduction.

[49] Masella AP (2012). PANDAseq: paired-end assembler for illumina sequences. BMC Bioinformatics.

[50] Edgar RC (2013). UPARSE: highly accurate OTU sequences from microbial amplicon reads. Nat Methods.

[51] Cole JR (2014). Ribosomal database project: data and tools for high throughput rRNA analysis. Nucleic Acids Res.

[52] Ravel J (2011). Vaginal microbiome of reproductive-age women. Proc Natl Acad Sci U S A.

[53] Caporaso JG (2010). QIIME allows analysis of high-throughput community sequencing data. Nat Methods.

[54] Segata N (2011). Metagenomic biomarker discovery and explanation. Genome Biol.

[55] Mohd Shaufi MA (2015). Deciphering chicken gut microbial dynamics based on high-throughput 16S rRNA metagenomics analyses. Gut Pathog.

[56] 1Institute for Systems Biology, S., Washington 98103, USA; 2Whitehead Institute for Biomedical Research, Cambridge, and U.D.o.B. Massachusetts 02142, University of California–San Diego, La Jolla, California 92093, USA, Cytoscape: A Software Environment for Integrated Models of Biomolecular Interaction Networks*.* Genome Research, 2003. 13:2498–2504(ISSN1088–9051 /03). 10.1101/gr.1239303 PMC403769 14597658

[57] Douglas GM, et al. PICRUSt2: An improved and extensible approach for metagenome inference. bioRxiv. 2019.

