# Supplementary figures and images for: Changes in the vaginal microbiota associated with primary ovarian failure
Source: BMC Microbiol. 2020 Jul 29;20:230. doi: 10.1186/s12866-020-01918-0 (PMC7392721; doi:10.1186/s12866-020-01918-0)

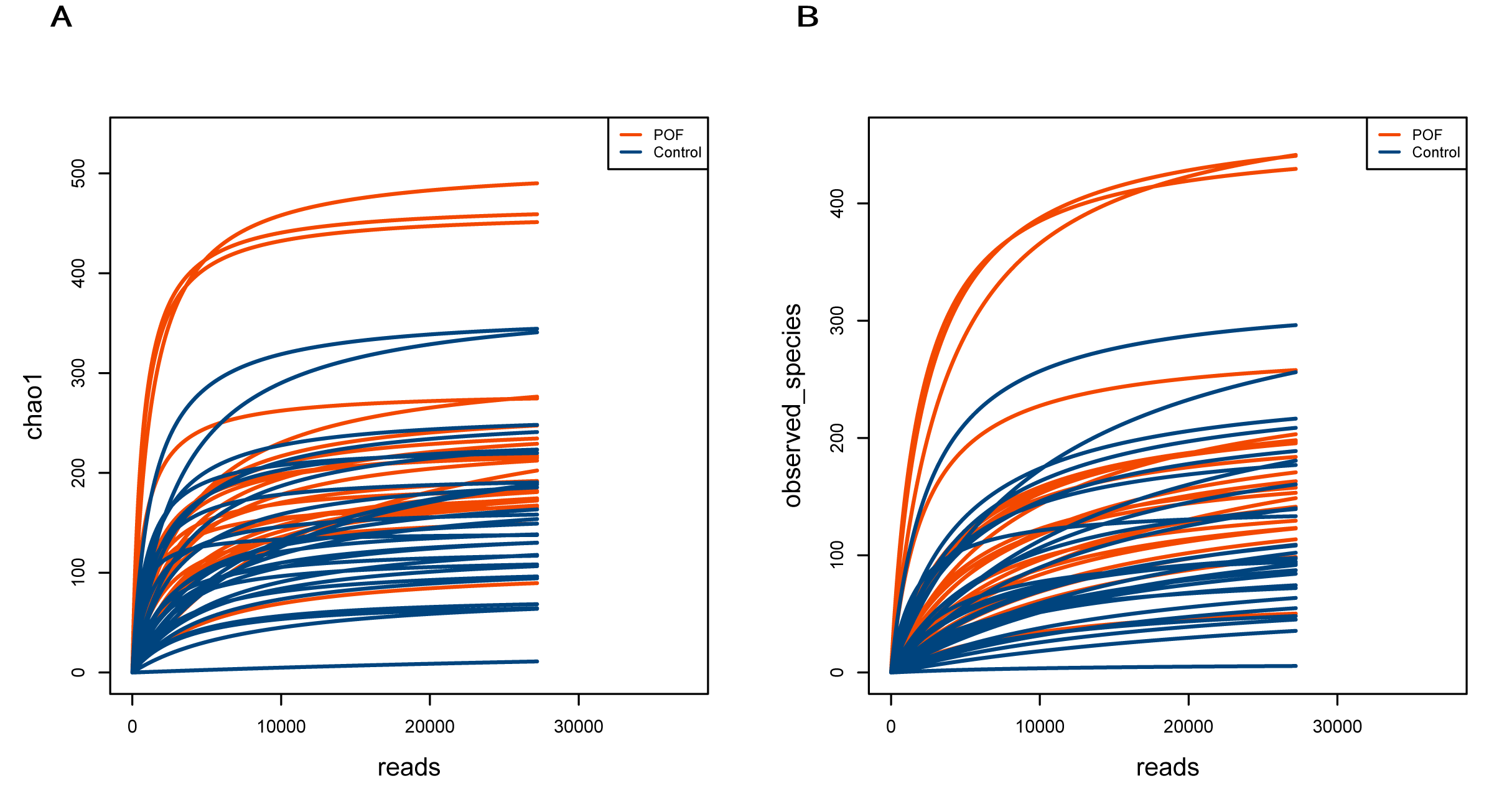

Supplement: Supplementary file 5 — Additional file 5: Figure S1. Diagram of the number of clean reads randomly selected from a sample, showing species diversity within each single sample. Sufficient sequencing depth of each sample is visualized by each of the curves becoming flat. Red indicates POF Groups; Blue indicates Control Group. [file 12866_2020_1918_MOESM5_ESM.tif]

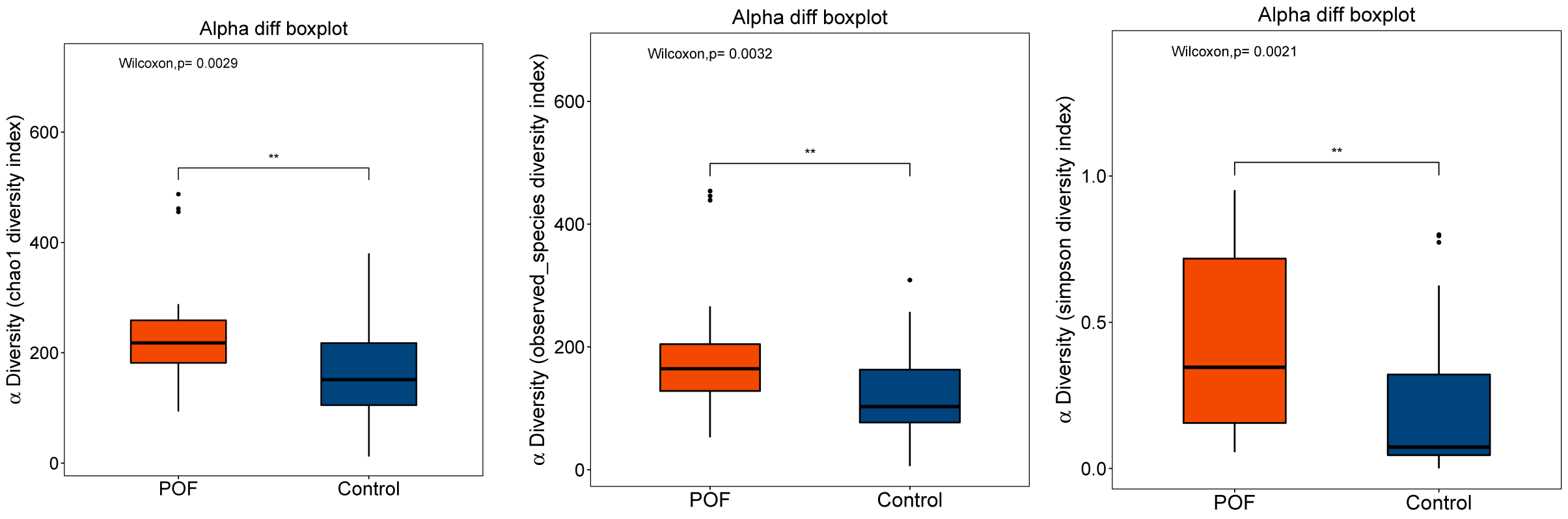

Supplement: Supplementary file 6 — Additional file 6: Figure S2. The abscissa indicates sample grouping, and the ordinate indicates the alpha diversity index value under different groupings. The Chao1 index was used to estimate the total number of OTUs contained within a sample. Observed_ indicates the actual number of OUT observed. Greater Simpson value, higher diversity. Key: *0.01 < p < 0.05, **p < 0.01, “NS” indicates no significant difference. [file 12866_2020_1918_MOESM6_ESM.tif]

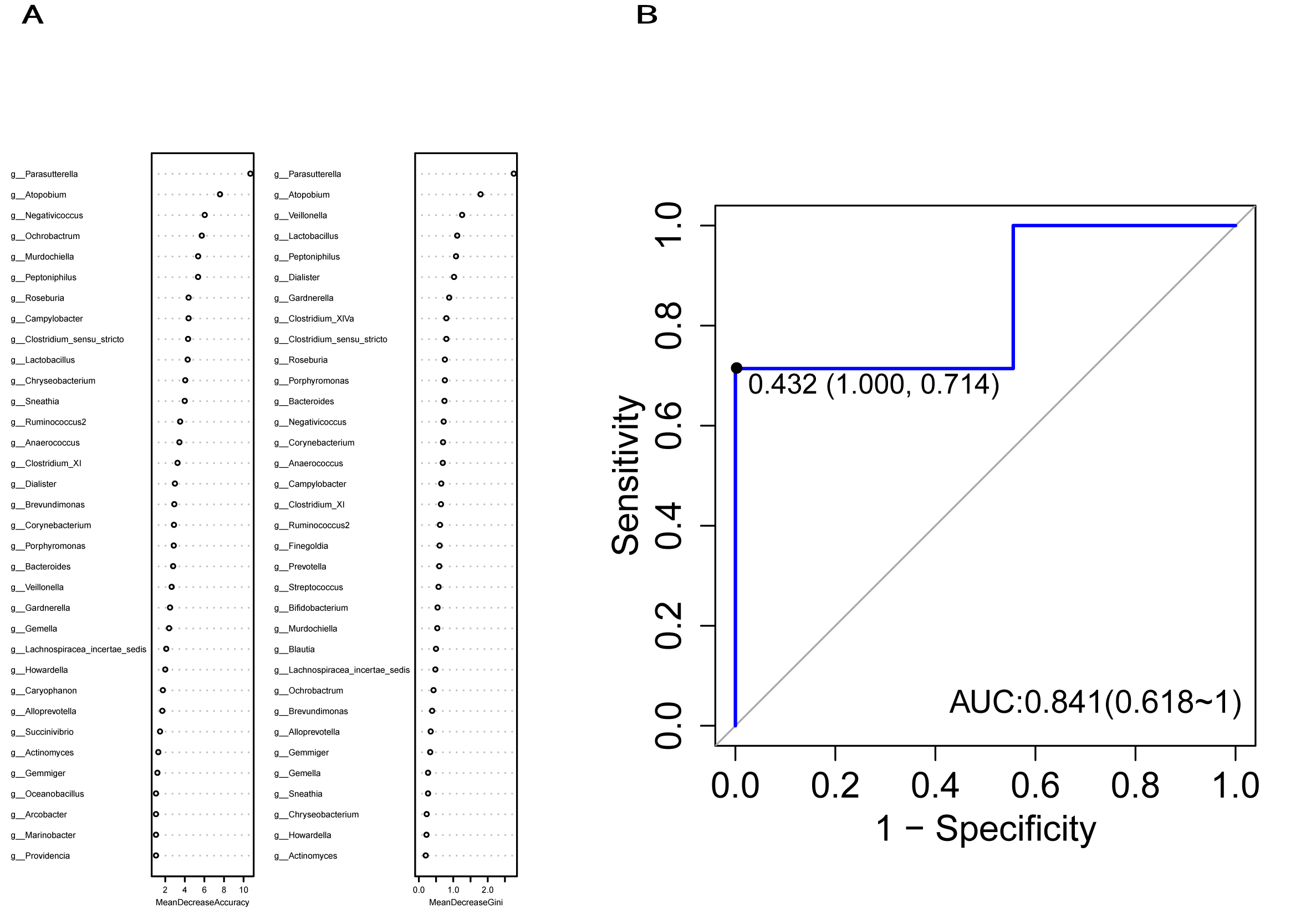

Supplement: Supplementary file 7 — Additional file 7: Figure S3. The predictive model based on genus-level abundance taxa using a random forests model. A: The difference in contributions of different species enabled groups A and B to be distinguished; B: The ROC curve of a random forest model was constructed based on the sorted different species, where the abscissa is 1-specificity and the ordinate is sensitivity. When the area under the curve (AUC is 0.5–0.7, the accuracy is low; when AUC is 0.7–0.9, there is certain accuracy; when AUC is above 0.9, the accuracy is high). Larger AUC indicates better model prediction effect. [file 12866_2020_1918_MOESM7_ESM.tif]

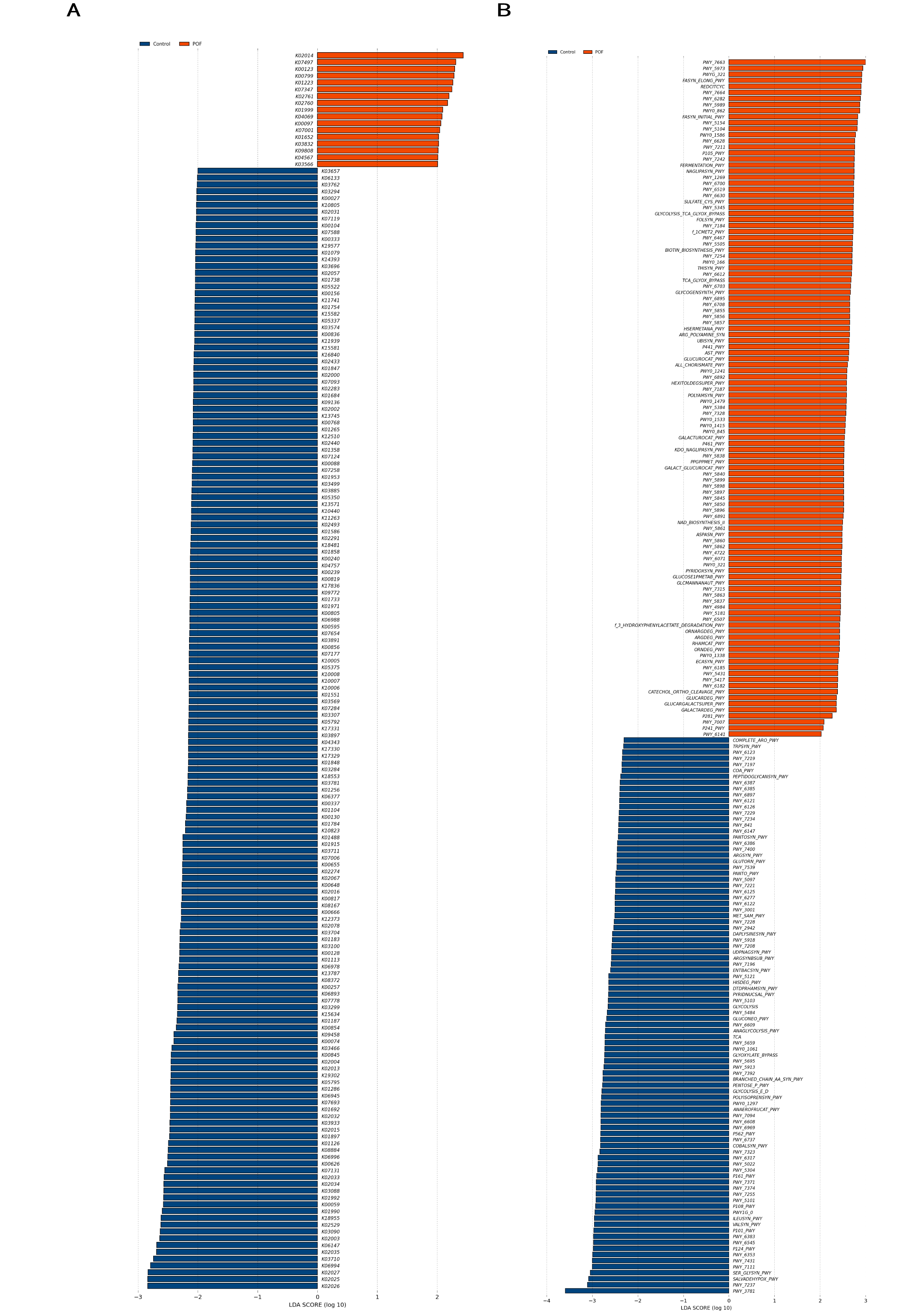

Supplement: Supplementary file 8 — Additional file 8: Figure S4. Functional predictions of vaginal flora of the POF and control groups. A: The abscissa is the log value obtained after KO has a significant effect in different groupings through LDA, the threshold for LDA was 2. Different colours represent that the EC is enriched in different groups of samples. B: The abscissa is the log value obtained after MetaCyc_pathway has a significant effect in different groupings through LDA, the threshold for LDA was 2. Different colours represent that the MetaCyc_pathway is enriched in different groups of samples. [file 12866_2020_1918_MOESM8_ESM.tif]

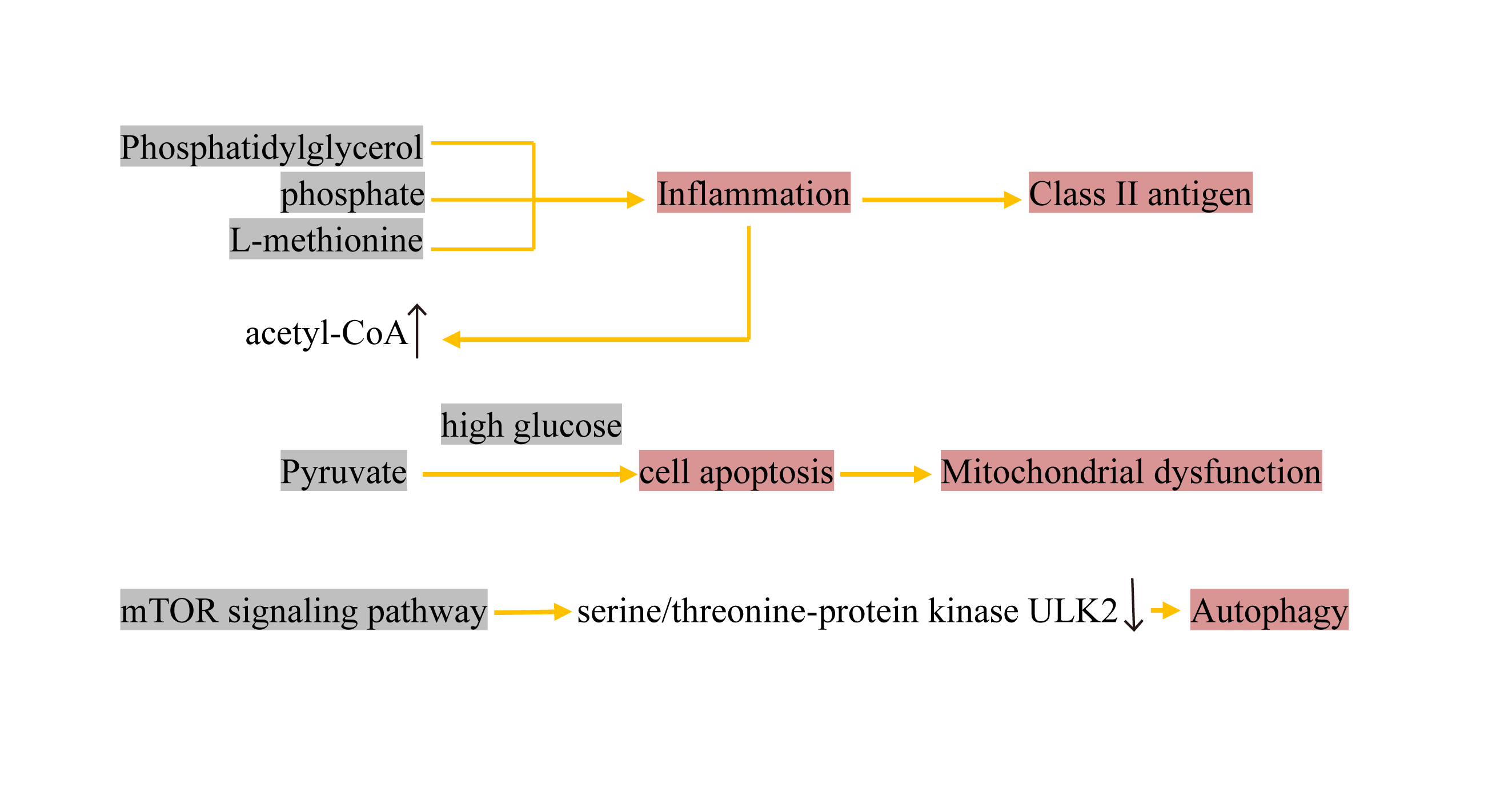

Supplement: Supplementary file 9 — Additional file 9: Figure S5. This diagram stands for a hypothesis regarding the possible mechanisms underlying relationship between vaginal microbiota abundance and pathological changes of POF. Gray text boxes denote enriched microbes, product, or pathway in POF patients. Red text boxes denote the pathological changes and complications in POF patients. [file 12866_2020_1918_MOESM9_ESM.tif]
